# Teaching and Assessing Professionalism in Medical Learners and Practicing Physicians*

**DOI:** 10.5041/RMMJ.10195

**Published:** 2015-04-29

**Authors:** Paul S. Mueller

**Affiliations:** Consultant, Division of General Internal Medicine, Mayo Clinic, Rochester, MN, USA; Professor of Medicine and Professor of Biomedical Ethics at the Mayo Clinic College of Medicine, Rochester, MN, USA; Associate Editor of NEJM Journal Watch General Medicine

**Keywords:** Assessment, ethics, medical education, medical professionalism, professionalism

## Abstract

Professionalism is a core competency of physicians. Clinical knowledge and skills (and their maintenance and improvement), good communication skills, and sound understanding of ethics constitute the foundation of professionalism. Rising from this foundation are behaviors and attributes of professionalism: accountability, altruism, excellence, and humanism, the capstone of which is professionalism. Patients, medical societies, and accrediting organizations expect physicians to be professional. Furthermore, professionalism is associated with better clinical outcomes. Hence, medical learners and practicing physicians should be taught and assessed for professionalism. A number of methods can be used to teach professionalism (e.g. didactic lectures, web-based modules, role modeling, reflection, interactive methods, etc.). Because of the nature of professionalism, no single tool for assessing it among medical learners and practicing physicians exists. Instead, multiple assessment tools must be used (e.g. multi-source feedback using 360-degree reviews, patient feedback, critical incident reports, etc.). Data should be gathered continuously throughout an individual’s career. For the individual learner or practicing physician, data generated by these tools can be used to create a “professionalism portfolio,” the totality of which represents a picture of the individual’s professionalism. This portfolio in turn can be used for formative and summative feedback. Data from professionalism assessments can also be used for developing professionalism curricula and generating research hypotheses. Health care leaders should support teaching and assessing professionalism at all levels of learning and practice and promote learning environments and institutional cultures that are consistent with professionalism precepts.

## INTRODUCTION

The practice of medicine is an art, not a trade, a calling, not a business—a calling in which your heart will be exercised equally with your head.

(William Osler^1^)

In a well-arranged community, a citizen should feel that he can at any time command the services of a [doctor] who has received a fair training in the science and art of medicine, into whose hands he may commit with safety the lives of those near and dear to him.

(William Osler^2^)

Imagine you are in an unfamiliar city—as a tourist, a business traveler, etc.—and you suddenly experience chest pain, light-headedness, and sweats. Worried, you call “911,” emergency medical technicians arrive, and you are taken by ambulance to the nearest hospital. You know nothing about the hospital, its physicians, or its staff. What attributes and behaviors do you desire and expect in the physician who will be caring for you? Most people would have the same expectations as expressed by Osler: a competent and trustworthy physician who is called to act for the benefit of patients and manifests accountability, altruism, excellence, and accountability—a physician who manifests professionalism.

## WHAT IS PROFESSIONALISM?

The word *profession* derives from the Latin *professio*, or public declaration.^3^ A *profession* is “a calling requiring specialized knowledge and often long and intensive preparation including instruction in skills and methods as well as in the scientific, historical, or scholarly principles underlying such skills and methods, maintaining by force of organization or concerted opinion high standards of achievement and conduct, and committing its members to continued study and a kind of work which has for its prime purpose the rendering of a public service.”^3^ Medicine is a *profession*.

The attributes, behaviors, commitments, values, and goals that characterize a profession constitute *professionalism*, and its members are *professionals*.

However, professionalism is an abstract concept. Definitions used by medical societies typically list attributes and behaviors associated with professionalism. For example, the Accreditation Council for Graduate Medical Education (ACGME) lists “professionalism” as one of six core competencies that physicians-in-training (i.e. residents and fellows) must possess before graduating from their training programs.^4^ The ACGME’s definition of professionalism includes a list of attributes and behaviors such as accountability, altruism, commitment to excellence, compassion, integrity, respect, responsiveness, sensitivity to diversity, and sound ethics.^5^ Calling professionalism the “foundation of the social contract for medicine,” the American Board of Internal Medicine Foundation, the American College of Physicians–American Society of Internal Medicine Foundation, and the European Federation of Internal Medicine, in the “Physician Charter,” list three “fundamental principles” and 10 “professional responsibilities” that characterize professionalism (Table 1).^6^,^7^

**Table 1.** The Physician Charter on Medical Professionalism^6^,^7^ (used with the permission of the American College of Physicians).

Fundamental Principles
➢ Principle of primacy of patient welfare ➢ Principle of patient autonomy ➢ Principle of social justice
Professional Responsibilities ➢ Commitment to professional competence ➢ Commitment to honesty with patients ➢ Commitment to patient confidentiality ➢ Commitment to maintaining appropriate relations with patients ➢ Commitment to improving quality of care ➢ Commitment to improving access to care ➢ Commitment to a just distribution of finite resources ➢ Commitment to scientific knowledge ➢ Commitment to maintaining trust by managing conflicts of interests ➢ Commitment to professional responsibilities

Going further, the American Board of Medical Specialties, which represents 24 specialties, asserts that professionalism transcends lists of desired attributes and behaviors:

Medical professionalism is a [normative] belief system about how best to organize and deliver health care, which calls on group members to jointly declare (“profess”) what the public and individual patients can expect regarding shared competency standards and ethical values and to implement trustworthy means to ensure that all medical professionals live up to these promises.^8^

In other words, professionalism is the reason medical learners and practicing physicians should manifest the aforementioned desired attributes and behaviors.

Overall, definitions of professionalism underscore the importance of scientific, procedural, interpersonal, and ethical competencies; these competencies are equally important (e.g. being only knowledgeable and skillful is insufficient for the medical professional).^8^ These definitions also underscore the physician’s *fiduciary* duties to the patient. An ill or injured patient is inherently vulnerable. In contrast, a physician has specialized knowledge and skills, access to diagnostic and therapeutic interventions (e.g. prescribing privileges), and other privileges that most patients lack. Hence, a patient must trust his or her physician is acting in the patient’s interest. Indeed, trust is an essential feature of the physician–patient relationship.^9^

Society expects physicians will be competent, skillful, ethical, humanistic, altruistic, and trustworthy—professional—and that physicians and the medical profession will promote individuals’ and the public’s health and well-being. In exchange, society allows the medical profession to be autonomous (i.e. autonomy to admit, train, graduate, certify, monitor, discipline, and expel its members) and provides means to meet its responsibilities (e.g. infrastructure, subsidization of training and research programs, etc.).^6^,^10^,^11^ The relationship between the medical profession and society—the “social contract”—is formalized through licensure.

## A FRAMEWORK FOR PROFESSIONALISM

**Figure 1.**
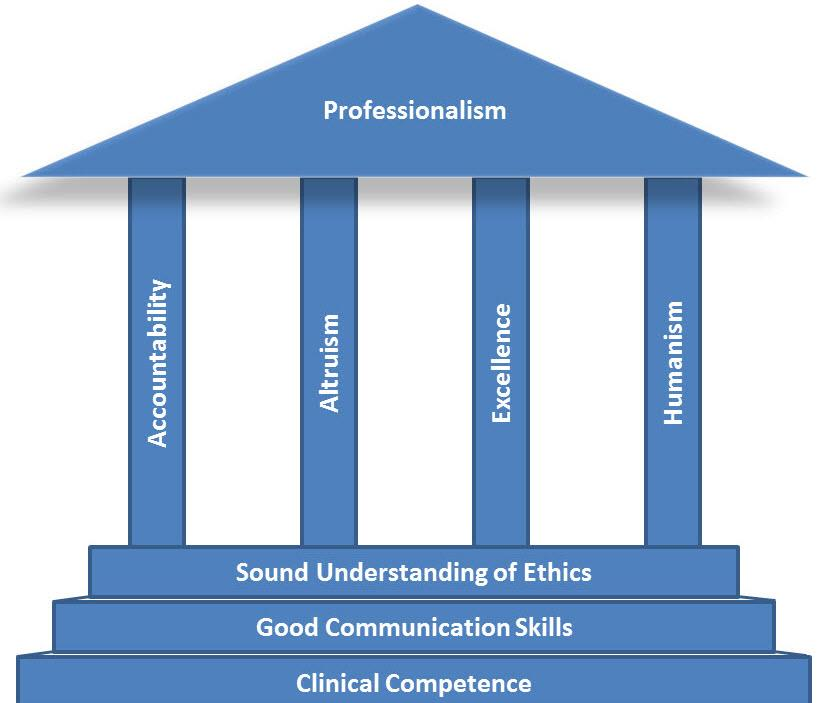
**A Framework for Professionalism.** Modified with the permission of *The Keio Journal of Medicine*.^33^,^76^

Arnold and Stern have proposed a framework for professionalism (Figure 1).^12^ The foundation of this framework is clinical competence, effective communication skills, and a sound understanding of ethics. Being a physician requires specialized knowledge and skills that require continuous maintenance and good communication skills. Physicians—regardless of specialty—must be able to discern patients’ health care-related concerns, goals, and preferences and work in multidisciplinary teams (e.g. teams comprising other physicians, nurses, physical therapists, pharmacists, social workers, learners, etc.); these tasks require good communication skills. Being a physician also requires a sound understanding of ethics. Because of the nature of their work, physicians inevitably encounter ethical dilemmas (e.g. requests to withdraw life-prolonging treatments from patients who lack decision-making capacity, medical futility, duty to care during epidemics, etc.).

Built on this foundation are key attributes—or pillars—of professionalism: accountability (the physician [and the profession] takes responsibility for his or her behaviors and actions), altruism (patients’ interests, not physicians’ [or the profession’s] self-interests, guide physicians’ behaviors and actions), excellence (the physician commits to continuous maintenance of knowledge and skills, lifelong learning, and the advancement of knowledge), and humanism (compassion, empathy, integrity, and respect). The totality of the framework—or capstone—is professionalism.^12^ “Being a physician—taking on the identity of a true professional—also involves a number of value orientations, including a general commitment not only to learning and excellence of skills but also to behavior and practices that are authentically caring.”^11^ As implied by Osler, the goal is to have competent and trustworthy physicians who have internalized and manifest attributes of professionalism.

## WHY IS PROFESSIONALISM IMPORTANT?

The aforementioned definitions and framework notwithstanding, there are a number of reasons why professionalism among medical learners and practicing physicians is important (Box 1).

Box 1. **Reasons Why Professionalism among Medical Learners and Practicing Physicians is Important**

➢ Patients expect physicians to be professional
➢ Medical societies and accrediting organizations expect physicians to be professional
➢ Professionalism is associated with improved medical outcomes
➢ There is a “business case” for professionalism

### Patients Expect Their Physicians to Be Professional

In a study^13^ at Mayo Clinic (the author’s institution), about 200 randomly selected patients seen in 14 different specialties were interviewed by phone. The patients were asked to describe their best and worst experiences with a physician. From these data, a list of seven ideal physician behaviors was generated: being confident, empathetic (“understands my feelings”), forthright (“tells me what I need to know”), humane (kind and compassionate), methodical, personal (i.e. regarding the patient as a human being, not as a disease), and respectful. Obviously, most patients do not want physicians who manifest opposite behaviors such being deceptive, hurried and haphazard, cold and callous, and disrespectful^14^—behaviors that are contrary to the precepts of professionalism.

Other studies have shown that willingness to recommend is associated with professionalism. In a study involving more than 23,000 inpatients, patients undergoing outpatient procedures, and patients receiving emergency care, compassion provided to patients had the strongest association with patients’ willingness to recommend.^15^ In a study involving more than 2,000 patients with cancer, key drivers of perceived service quality associated with willingness to recommend were “team helping you understand your medical condition,” “staff genuinely caring for you as an individual,” and “whole person approach to patient care.”^16^ In another study involving more than 33,000 patients cared for at 131 hospitals, the strongest predictors of willingness to recommend were interpersonal aspects of care such as physician and nurse behaviors (e.g. “Doctors showed courtesy” and “Nurses showed courtesy and respect”).^17^ Similarly, internal surveys conducted at Mayo Clinic have shown that high patient ratings of quality of care and satisfaction are associated with physician behaviors that manifest professionalism: having a caring attitude, listening, providing adequate explanations (e.g. of diagnoses, test results, and treatment plans), being thorough and efficient, and projecting a sense of teamwork among the health care team.

### Medical Societies and Accrediting Organizations Expect Physicians to be Professional

As mentioned previously, the ACGME lists “professionalism, as manifested through a commitment to carrying out professional responsibilities, adherence to ethical principles, and sensitivity to a diverse patient population” as a core competency (along with patient care, medical knowledge, practice-based learning and improvement, systems-based practice, and interpersonal skills and communication).^4^ Within 15 months of its release, the “Physician Charter” (Table 1) was endorsed by 90 specialty societies.^7^ The American Board of Internal Medicine’s certification program has ethics and professionalism content.^18^ The Joint Commission, a non-profit organization that accredits US health care institutions, requires institutions to have processes in place for addressing ethical concerns that arise while caring for patients; has standards that define acceptable physician and allied health care provider behaviors; directs institutions to create and implement processes for addressing unprofessional physician and allied health care provider behaviors; and recommends that institutions teach and assess professionalism in health care providers.^19^,^20^

### Professionalism is Associated with Improved Medical Outcomes

Professionalism is associated with increased patient satisfaction, trust, and adherence to treatment plans; fewer patient complaints; and reduced risk for of litigation.^9^,^21^,^22^ Effective communication is associated with improved patient outcomes including satisfaction, symptom control, physiologic measures (e.g. blood pressure), emotional health, and adherence to treatment plans.^9^,^23^ Effective communication ensures safe and appropriate care and may prevent avoidable adverse medical events.^24^ Professionalism is associated with physician excellence including medical knowledge, skills, and conscientious behaviors.^5^,^21^,^25^ Indeed, unprofessional behavior and clinical excellence rarely coexist.^21^

Unfortunately, unprofessional and disruptive physician behaviors are common. In a survey of more than 1,600 physician leaders, 95% reported they had routinely dealt with unprofessional and disruptive physician behaviors including insults, yelling, disrespect, abuse, and refusal to carry out duties; these behaviors involved patients, nurses, other physicians, and administrators.^26^ Other studies have shown that most nurses and physicians have observed or experienced unprofessional and disruptive physician behaviors.^27^–^30^ Physician abuse of trainees and pharmacists is also common.^31^,^32^

These data are important because unprofessional and disruptive physician behaviors are associated with reduced patient satisfaction, increased patient complaints, and increased risk for of litigation.^9^,^21^,^22^ These behaviors also result in reduced communication, efficiency, productivity, learner and nurse satisfaction, and teamwork along with higher employee turnover, costs, and learner burnout and depression.^21^,^22^,^33^ In a study involving the perioperative setting, unprofessional and disruptive physician behaviors not only increased levels of stress and frustration, impaired concentration and communication, and negatively affected teamwork, but were also perceived to increase risk for adverse events and compromise patient safety.^34^ The Joint Commission estimates that 60% of avoidable adverse events are due to communication errors.^24^ Hence, allowing unprofessional and disruptive physician behaviors to persist may compromise patient safety.^9^ Furthermore, left unaddressed, unprofessional and disruptive behavior may eventually come to be regarded by some medical learners and other practicing physicians as ordinary, and in turn they may manifest such behavior themselves (i.e. negative role modeling).^35^,^36^

Notably, prior research has shown that physicians disciplined by state medical boards had higher likelihood of manifesting unprofessional behaviors during medical school (e.g. poor initiative and motivation, poor reliability and responsibility, and lack of adaptability and self-improvement) compared to non-disciplined physicians.^21^,^37^,^38^ These findings highlight the importance of monitoring for unprofessional behaviors in medical learners and practicing physicians and remediating such behaviors when observed. Doing so sends a strong message to patients, medical learners, practicing physicians, and society regarding the importance of professionalism and fulfills the medical profession’s obligation of self-regulation.

Evidence suggests that institutional professionalism is also associated with improved medical outcomes. Recall the case scenario at the beginning of this article: you are being taken to the nearest hospital because of acute chest pain. One possible cause is acute myocardial infarction. In a recent study that compared US hospitals ranked in the top 5% in mortality rates for patients with acute myocardial infarction with hospitals in the bottom 5%, evidence-based protocols and processes for acute myocardial infarction care did not distinguish high-performing from low-performing hospitals. However, high-performing hospitals were characterized by organizational cultures that promoted efforts to improve acute myocardial infarction care (e.g. staff expressed shared values of providing high-quality care; senior leadership demonstrated unwavering commitment to high-quality care; presence of physician champions and empowered nurses; strong communication and co-ordination; and effective problem-solving and organizational learning).^39^

### There Is a “Business Case” for Professionalism

In the US and elsewhere, many health care institutions and systems compete with each other for patients. Most institutions and systems have highly trained physicians, nurses, and staff, up-to-date diagnostic and therapeutic equipment, and good facilities. Yet, some institutions and systems attract more patients than others, including patients from all over the world—patients who are motivated to travel at great expense and inconvenience to these institutions. For example, Mayo Clinic’s largest facility, located in Rochester, Minnesota, which is a city of only 120,000 inhabitants about 100 kilometers from the nearest large metropolitan area (Minneapolis-St. Paul), attracts hundreds of thousands of patients seeking medical care annually. Why?

The most striking thing about Mayo is the impact everywhere of its primary value, namely that “the needs of the patient come first.” This is no fly-by-night mission statement. On the contrary it is the single point of focus in everything Mayo does, pursued from the Clinic’s earliest days with almost religious fervor. You see it in clinical practice, the attitude of staff, the management ethos, the design of buildings, the patient-centered focus in medical education and research, even in the dress code for staff. Patients feel it for themselves. That simple primary value epitomizes the culture. It is at the heart of the Mayo Clinic Model of Care.^40^

Mayo Clinic’s primary value, “the needs of the patient come first” (which is derived from a speech given by Dr William Mayo at the 1910 Rush Medical College commencement^41^), and the “Mayo Clinic Model of Care”^42^ (Table 2) are manifestations of an institutional culture that promotes and fosters professionalism and key factors in drawing an international population of patients to a remote place in the middle of North America. Correspondingly, as a non-profit institution, Mayo Clinic has consistently maintained positive net operating income and impressive philanthropic support while engaged in extensive clinical practice, education, and research endeavors.^43^,^44^ Hence, there is a “business case” for professionalism.

**Table 2.** The Mayo Clinic Model of Care (used with the permission of the Mayo Foundation).^42^

**Patient Care**
Collegial, co-operative, staff teamwork with true multi-specialty integration An unhurried examination with time to listen to the patient Physicians taking personal responsibility for directing patient care over time in a partnership with the local physician Highest-quality patient care provided with compassion and trust Respect for the patient, family, and the patient's local physician Comprehensive evaluation with timely, efficient assessment and treatment Availability of the most advanced, innovative diagnostic and therapeutic technology and techniques
**The Mayo Environment**
Highest-quality staff mentored in the culture of Mayo and valued for their contributions Valued professional allied health staff with a strong work ethic, special expertise, and devotion to Mayo A scholarly environment of research and education Physician leadership Integrated medical record with common support services for all outpatients and inpatients Professional compensation that allows a focus on quality, not quantity Unique professional dress, decorum, and facilities

However, this case is not confined to Mayo Clinic. For example, as mentioned previously, prior research has shown that professionalism is associated with patient willingness to recommend in various settings.^15^,^17^ In an environment that is highly competitive, individual physician and institutional professionalism are not just good things, but are necessary for survival. In contrast, unprofessional and disruptive physician behaviors can negatively affect patient willingness to recommend.^22^

## TEACHING AND ASSESSING PROFESSIONALISM SHOULD NOT BE LEFT TO CHANCE ALONE

Historically, it has been assumed that medical learners will learn, and practicing physicians will manifest, the precepts, attributes, and behaviors of professionalism. However, in recent years the medical profession has been criticized for perceived and real breaches of professionalism (e.g. inappropriate behaviors,^45^ violations of online professionalism,^46^ and financial conflicts of interest^9^,^47^,^48^). In response, health care institutions, medical societies, and accrediting organizations have encouraged and required teaching, assessing, and promoting professionalism (see above). Indeed, teaching and assessing professionalism do not occur by chance alone. In order for medical learners and practicing physicians to be professional, the foundational elements of professionalism (e.g. communication skills and ethics) and the attributes of professionalism—accountability, altruism, excellence, and humanism—should be intentionally taught. In addition, professionalism should be intentionally assessed. Assessment motivates individuals to learn and adhere to professionalism precepts and determines whether competency in professionalism has been achieved.^49^

## HOW SHOULD PROFESSIONALISM BE TAUGHT?

Cruess and Cruess have articulated principles for teaching professionalism (Box 2).^50^,^51^ First, institutional leaders (e.g. CEOs, deans, department chairs, etc.) should authentically and publicly support teaching professionalism. Such support should include adequate resources (e.g. time for teachers); providing adequate resources conveys the message that professionalism is important and ensures a program’s success. Second, the “cognitive base” of professionalism (e.g. historical roots, definition, values, attributes, behaviors, and associated responsibilities) should be explicitly taught. Third, learning environments should align with the institution’s mission statement and professionalism precepts. Because behaviors of institutions can affect the behaviors of individual medical learners and physicians, institutions should manifest organizational professionalism competencies including accountability, fairness, integrity, respect, and service.^52^ In other words, it does not make sense to teach (or assess) professionalism in learning environments that are inimical to its principles and commitments.^11^ Fourth, faculty members responsible for teaching professionalism should be highly respected colleagues (i.e. those who manifest attributes and behaviors of professionalism) who have direct access to institutional leadership. Finally, all faculty members should be familiar with the “cognitive base” of professionalism and provided methods for teaching and assessing professionalism in their respective settings. The overarching goal is for medical learners and practicing physicians to internalize professionalism precepts.^50^

Box 2. **Not to Be Left to Chance Alone: Principles for Teaching Professionalism.^50^,^51^**

➢ Institutional leaders should authentically and publicly support teaching professionalism; such support should include adequate resources (e.g. time for teachers)
➢ The “cognitive base” of professionalism (e.g. historical roots, definition, values, attributes, behaviors, and associated duties and responsibilities) should be explicitly taught
➢ Learning environments should align with the institution’s mission statement and professionalism precepts
➢ Faculty members responsible for teaching professionalism should be highly respected colleagues who have direct access to institutional leadership
➢ All faculty members should be familiar with the “cognitive base” of professionalism and provided methods for teaching professionalism (e.g. lectures, role modeling, reflection, and interactive sessions) in their respective settings

Various methods for teaching professionalism are available. The didactic lecture is a popular and efficient means of summarizing large amounts of information and can improve knowledge and change attitudes (e.g. the importance, or “cognitive base,” of professionalism).^53^,^54^ However, teaching programs that rely primarily on didactic lectures do not necessarily improve patient outcomes or physician performance.^55^–^59^ Teaching and learning during didactic lectures can be enhanced with audio and video (e.g. showing examples of professional and unprofessional behaviors) and using an audience response system (e.g. presenting a clinical ethical dilemma scenario and offering the audience a list of responses to the dilemma). Audience response systems allow the teacher to gauge the audience’s knowledge and then adjust his or her teaching accordingly.

Web-based teaching modules are growing in popularity and have multiple advantages over didactic lectures. In addition to summarizing large amounts of information, web-based modules can be accessed when convenient for learners (including at the point of care^60^), disseminated to large numbers of learners, and coupled with assessments that determine whether learners have mastered the content. Teaching and learning during web-based modules can be enhanced with audio and video. “Pop-up” multiple-choice questions can be embedded in web-based modules. The learner chooses an answer and, based on the result, the module congratulates the learner on being correct or provides the correct answer with an explanation. Self-assessment questions have been shown to enhance learning; however, the number of questions per module should be limited.^61^ Web-based teaching modules can also incorporate online discussions among teachers and learners. Nonetheless, whether web-based teaching modules are effective for teaching professionalism is unknown. Overall, relying on didactic lectures and web-based modules alone for teaching professionalism is likely insufficient for learning.

A consensus is emerging that role modeling is an effective means of teaching professionalism.^21^,^50^,^51^,^62^,^63^ Ideal role models manifest clinical competence, excellent teaching skills, and desirable personal qualities and are capable of demonstrating the attributes and behaviors of professionalism during interactions with patients, medical learners, colleagues, allied health care staff, and teams. Role models’ actions should be consistent with formal professionalism curricula.^21^ Experience suggests that learners watch, embrace, and mimic attitudes and behaviors of role models. Teachers should leverage this phenomenon by using interactions with patients, colleagues, and health care team members as opportunities to role model ideal attributes and behaviors (i.e. accountability, altruism, excellence, and humanism). Likewise, institutions should embrace and foster a culture of professionalism.^52^ Without institutional cultures and learning environments that support and promote professionalism, professionalism curricula may be viewed as inauthentic and role models’ efforts thwarted.

Role modeling can be enhanced with reflection.^62^,^63^ For example, teachers may ask medical learners to reflect on meaningful events as they occur while caring for patients (e.g. difficult diagnosis, communication failure, adverse event, ethical dilemma, etc.). Teachers who role model behaviors (e.g. delivering “sad, bad, or unexpected news” to patients and loved ones) should ask learners to reflect on, and engage learners in discussions regarding, the role modeled behaviors (e.g. “What went well?” and “What could have been done better?”).

Experiential and interactive teaching methods such as case discussions and hands-on practice sessions can improve learner performance and patient outcomes.^55^–^59^,^64^,^65^ These methods can be applied to teaching professionalism and how to recognize and address professionalism challenges. Examples include discussion groups (e.g. the “challenging case”), role play (e.g. “speaking up” regarding an impaired colleague), simulation (e.g. giving “sad, bad, or unexpected news” to a patient),^64^ team-based learning,^65^ and self-reflection (e.g. reflecting on actions during an event through journaling or discussions with a peer, colleague, or mentor).^62^,^66^ The “critical incident report,” which is a short narrative written by an individual describing a meaningful patient care event, can be an effective tool for teaching professionalism, especially if used with self-reflection and group discussion.^67^ These methods should be used in safe and structured environments (e.g. facilitated by a respected faculty member).^50^

Teaching and learning professionalism can be further enhanced by various means.^62^,^68^ Professionalism curricula should be relevant; learners will be more engaged in learning professionalism when it is taught in the context of their specialty (e.g. obstetrics and gynecology learners should learn about professionalism in the context of obstetrics and gynecology practice). Professionalism curricula should also be practical (e.g. obstetrics and gynecology learners should learn how to identify and address professionalism challenges in obstetrics and gynecology practice). Professionalism curricula should challenge and facilitate growth of communication skills. The hidden curriculum, “influences that function at the level of organizational structure and culture,”^69^ should be characterized and addressed if it conflicts with institutional goals and formal curricula.^11^,^50^ For example, curricula that promote “speaking up” when patients are at risk for harm should be supported by an institutional culture that supports speaking up without threat of retaliation.^70^ Failure to address an adverse “hidden curriculum” conveys the message the institution’s goal to support and promote professionalism is not authentic. Finally, negative and disruptive role models should be removed from teaching roles.^5^,^36^

## HOW SHOULD PROFESSIONALISM BE ASSESSED?

Principles for assessing professionalism are listed in Table 3. As with any subject, the reason for assessing professionalism is to determine whether medical learners acquire, and practicing physicians have, this core competency. Assessment drives learning; medical learners will attempt to master a subject if they know they will be assessed regarding it (i.e. “They don’t respect what you expect; they respect what you inspect”^71^). Furthermore, assessing professionalism conveys the message to patients, medical learners, practicing physicians, the public, and others that professionalism is important and valued.^50^

**Table 3.** Principles for Assessing Professionalism in Medical Learners and Practicing Physicians (based on Table 4 of Mueller, 2009^33^ with permission of *The Keio Journal of Medicine*).

Multiple assessment tools should be used: ➢ Tests of knowledge (i.e. “cognitive base”) ➢ Multi-source 360-degree reviews (e.g. by faculty members, peers, allied health care staff [e.g. nurses], and others) ➢ Objective structured clinical examinations ➢ Patient assessments ➢ Simulation ➢ Critical incident reports ➢ Reviews of patient complaints and professionalism lapses
Individuals should know they are being assessed for professionalism
Commence at the start and continue throughout individuals’ careers; all levels of the medical hierarchy should be assessed
Assessments should be relevant to the individual’s level of education and specialty setting
Use collected data for formative and summative feedback and professionalism “portfolios”

Consistent with Arnold and Stern’s framework^12^ are the results of a recent systematic review,^72^ in which five clusters of professionalism suitable for assessment were identified: commitment to maintaining and improving competence in oneself, others, and systems; adherence to ethical principles; effective interactions with patients and their loved ones; effective interactions with colleagues, allied health care team members, and others within the health care system; and reliability and accountability. In turn, nine clusters of assessment tools were identified: paper-based tests, observed clinical encounters, patients’ opinions, supervisor reports, self-administered rating scales, collated views of coworkers, records of professionalism lapses, critical incident reports, and simulation. Indeed, because of the nature of professionalism, no single tool for assessing professionalism exists. Instead, multiple assessment tools must be used.^73^,^74^

An important tool for assessing professionalism is multi-source feedback using 360-degree reviews. Using this tool is feasible in medical learners^25^ and feasible, reliable, and valid in practicing physicians.^75^ Observers should include learners, peers, nurses, and other allied health care staff as each will have different perspectives of the individual’s professionalism. If possible, observations should be made in a variety of settings (e.g. outpatient clinic, inpatient ward, etc.) in which the individual works. Gathering data from multiple observers in varied settings helps ensure the observations are valid.^5^,^76^ As “most practicing physicians observe each other’s behaviors only in the hallways and conference rooms—rarely with patients,”^76^ efforts should be made to gather data regarding medical learners’ and practicing physicians’ behaviors when working with patients (e.g. from other learners, colleagues, nurses, and allied health staff). The identities of individuals participating in 360-degree reviews should be kept confidential, and institutions should have policies that prohibit retaliation related to such reviews.

Another tool for assessing professionalism among medical learners and practicing physicians is patient feedback. Patients can be surveyed at the point of care or at a later date. In a systematic review, 12 of 15 studies (80%) showed improved educational outcomes in physicians as a result of patient feedback. All studies that assessed Fitzpatrick level 1 (valuation), level 2 (learning), and level 3 (intended behavior) outcomes demonstrated positive results. However, only four of seven studies that assessed level 4 (change in actual performance or results) demonstrated positive results.^77^ According to the study authors, these discrepant results might be due to lack of precision in assessing actual performance, under-reporting of poor experiences by patients, and a true absence of effect of patient feedback. Future research should determine the reasons for the discrepant result. In the meantime, it is reasonable for institutions to develop and implement methods for improving performance in medical learners and practicing physicians who receive poor feedback from patients.

Other methods of assessing professionalism include objective structured clinical examinations,^5^,^73^ simulation,^78^ and “critical incident reports,”^79^ reviews of patient complaints and professionalism lapses, and tests of knowledge (i.e. of the “cognitive base”). A recent review describes these methods in detail.^74^ Notably, efforts to validate scores generated by professionalism assessment tools have lagged behind the creation of these tools. Future research should determine the validity of professionalism assessment tools.^80^

Professionalism assessments should be relevant to the individual’s level of education and specialty setting.^5^ Assessments should commence during medical school, be conducted regularly during residency and fellowship training, and continued throughout a physician’s career. Individuals should know they are being assessed for professionalism. Finally, observations should be made over a long period of time.^81^ The data generated by these multiple assessment tools can be used to create a “professionalism portfolio,” the totality of which should represent a picture of the individual’s professionalism.^82^ Portfolios can be used for formative feedback (i.e. feedback and action plans for improvement) and summative feedback (e.g. discipline individuals with unacceptable professionalism lapses). The data can also be used to reward exemplars.^5^,^76^ (Notably, the author, who is the chair of a division comprising about 90 faculty-level general internists, incorporates review of the faculty members’ “professionalism portfolio” into the annual review process, during which other data [e.g. the individual’s clinical productivity, teaching and research activities, career goals, etc.] are reviewed; similar portfolios are also used by training programs within the Mayo Clinic College of Medicine.^33^) Finally, the data can be used to develop and improve professionalism curricula and generate research hypotheses regarding professionalism in medical learners and practicing physicians.^5^,^73^

## Abbreviations:

ACGME: Accreditation Council for Graduate Medical Education.
